# Acetonitrile­[2-({bis[2,4,6-tris­(trifluorido­meth­yl)phen­yl]phosphan­yloxy}meth­yl)pyridine]­meth­ylpalladium(II) hexa­fluoro­anti­monate dichloro­methane hemisolvate

**DOI:** 10.1107/S1600536811005757

**Published:** 2011-02-23

**Authors:** Liuzhong Li, Peter S. White, Aiyou Hao

**Affiliations:** a School of Chemistry and Chemical Engineering, Shandong University, Jinan, 250100, People’s Republic of China; b Department of Chemistry, The University of North Carolina at Chapel Hill, Chapel Hill, North Carolina, 27599, USA

## Abstract

In the title compound, [Pd(CH_3_)(C_24_H_10_F_18_NOP)(CH_3_CN)][SbF_6_]·0.5CH_2_Cl_2_, the Pd^II^ atom has a distorted square-planar environment being coordinated by an acetonitrile N atom [Pd—N = 2.079 (3) Å], a methyl C atom [Pd—C = 2.047 (4) Å] and the bidentate ligand 2-({[2,4,6-tris­(trifluoro­meth­yl)phen­yl]phosphan­yloxy}meth­yl)pyridine (*L*). In *L*, the short distance of 3.621 (3) Å between the centroids of pyridine and benzene rings indicates the presence of a π–π inter­action. The crystal packing exhibits weak inter­molecular C—H⋯F contacts. The solvent mol­ecule has been treated as disordered between two positions of equal occupancy related by an inversion center.

## Related literature

For related compounds, see: Li *et al.* (2011 ▶) and references therein. Di[tris­(trifluoro­meth­yl)phen­yl]phosphine chloride was prepared according to Batsanov *et al.* (2002 ▶).
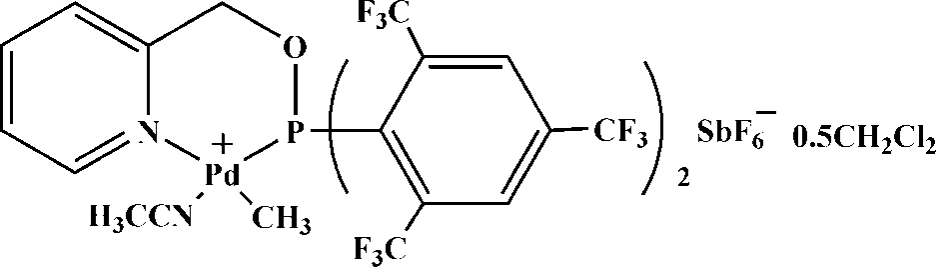


## Experimental

### 

#### Crystal data

[Pd(CH_3_)(C_24_H_10_F_18_NOP)(C_2_H_3_N)][SbF_6_]·0.5CH_2_Cl_2_
*M* *_r_* = 1142.00
Triclinic, 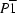

*a* = 8.6993 (4) Å
*b* = 11.8120 (5) Å
*c* = 18.1494 (8) Å
α = 78.557 (2)°
β = 82.007 (2)°
γ = 79.526 (2)°
*V* = 1787.14 (14) Å^3^
*Z* = 2
Cu *K*α radiation
μ = 12.64 mm^−1^
*T* = 100 K
0.38 × 0.13 × 0.11 mm

#### Data collection

Bruker APEXII CCD diffractometer
Absorption correction: numerical (*SADABS*; Bruker, 2007 ▶) *T*_min_ = 0.086, *T*_max_ = 0.348
19130 measured reflections
6422 independent reflections
5830 reflections with *I* > 2σ(*I*)
*R*_int_ = 0.035

#### Refinement

*R*[*F* ^2^ > 2σ(*F* ^2^)] = 0.036
*wR*(*F* ^2^) = 0.093
*S* = 1.05
6422 reflections
534 parameters
3 restraints
H-atom parameters constrained
Δρ_max_ = 1.46 e Å^−3^
Δρ_min_ = −0.88 e Å^−3^

### 

Data collection: *APEX2* (Bruker, 2007 ▶); cell refinement: *SAINT* (Bruker, 2007 ▶); data reduction: *SAINT*; program(s) used to solve structure: *SHELXS97* (Sheldrick, 2008 ▶); program(s) used to refine structure: *SHELXL97* (Sheldrick, 2008 ▶); molecular graphics: *SHELXTL* (Sheldrick, 2008 ▶); software used to prepare material for publication: *SHELXTL*.

## Supplementary Material

Crystal structure: contains datablocks I, global. DOI: 10.1107/S1600536811005757/cv5046sup1.cif

Structure factors: contains datablocks I. DOI: 10.1107/S1600536811005757/cv5046Isup2.hkl

Additional supplementary materials:  crystallographic information; 3D view; checkCIF report

## Figures and Tables

**Table 1:** Hydrogen-bond geometry (Å, °)

*D*—H⋯*A*	*D*—H	H⋯*A*	*D*⋯*A*	*D*—H⋯*A*
C4—H4*C*⋯F16^i^	0.98	2.44	3.310 (10)	148
C11—H11*B*⋯F11	0.99	2.58	3.489 (9)	154
C9—H9⋯F11	0.95	2.47	3.345 (9)	152

Symmetry code: (i) 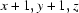.

**Fig. 1.**
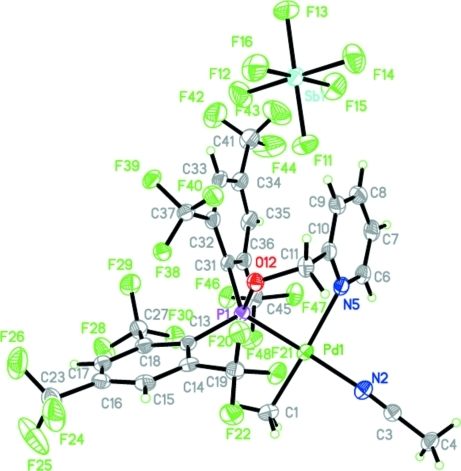
The molecular structure of (I) showing the atom-numbering scheme and 50% probabilty displacement ellipsoids. The H atoms and solvent molecules are omitted for clarity

## supplementary crystallographic information

### Comment

In continuation of our structural study of Pd complexes with phosphine-imine ligands (Li *et al.*, 2011), we present here the title compound (I). In (I) (Fig. 1), each Pd center has a distorted square-planar environment being coordinated by acetonitrile [Pd—N 2.079 (3) Å], methyl [Pd—C 2.047 (4) Å] and bidentate ligand *L*. The solvent molecule has been treated as disordered between two positions related by inversion center with occupancies fixed to 0.5 each. The crystal packing exhibits weak intermolecular C—H···F contacts (Table 1).

### Experimental

All manipulations of air- and/or moisture-sensitive compounds were conducted using standard Schlenk techniques. Argon was purified by passage through columns of BASF R3–11 catalyst (Chemalog) and 4Å molecular sieves. All solvents were deoxygenated, dried and distilled using common techniques. Di[tris(trifluoromethyl)phenyl]phosphine chloride were prepared according to the literature procedures(Batsanov *et al.*, 2002). A flame-dried Schlenk flask was charged with purified 2-pyridyl-carbinal (138 mg, 1.27 mmol) and dried THF (5 ml). The solution was cooled to -78°C and stirred for 30 min, 1.6 mol/l n-BuLi in hexane (0.8 ml, 1.28 mmol) was added slowly. After stirring of 1.0 hrs at -78°C, 800 mg of di[tris(trifluoromethyl)phenyl]phosphine chloride in THF(2 ml) was added slowly. Stirring for 1 day at -78°C, and brought it to room temperature and stirred overnight. 3.0 ml degassed saturated NaCl solution was charged for hydrolysis. After separation, dry and column purification, the ligand of 2- methoxy(di(2,4,6-tris(trifluoromethyl) phenyl)phosphino)] pyridine(0.45 g) was obtained. The yield is 50%. The neutral complex was prepared by reaction of the above ligand (1.0 equiv.) and (COD)PdMeCl (1.0 equiv.) at RT, and the cationic complex was obtained by reacting the neutral complex(1.0 equiv) with AgSbF_6_ (1.0 equiv.) at RT. Single crystal of the cationic complex was cultivated by recrystallization of CH_2_Cl_2_ and pentane. Anal. Calcd for C27H16F24N2OPPdSb: C, 29.49; H, 1.47; N, 2.55. Found: C, 29.52; H, 1.30; N, 2.27.

### Refinement

C-bound H atoms were geometrically positioned (C—H 0.95-0.99 Å) and refined as riding, with Uiso(H) = 1.2-1.5 Ueq(C).

### Crystal data

**Table:**

[Pd(CH_3_)(C_24_H_10_F_18_NOP)(C_2_H_3_N)][SbF_6_]·0.5CH_2_Cl_2_	*Z* = 2
*M**_r_* = 1142.00	*F*(000) = 1098
Triclinic, *P*1	*D*_x_ = 2.122 Mg m^−^^3^
Hall symbol: -P 1	Cu *K*α radiation, λ = 1.54178 Å
*a* = 8.6993 (4) Å	Cell parameters from 8055 reflections
*b* = 11.8120 (5) Å	θ = 2.5–69.1°
*c* = 18.1494 (8) Å	µ = 12.64 mm^−^^1^
α = 78.557 (2)°	*T* = 100 K
β = 82.007 (2)°	Block, colourless
γ = 79.526 (2)°	0.38 × 0.13 × 0.11 mm
*V* = 1787.14 (14) Å^3^	

### Data collection

**Table:**

Bruker APEXII CCD diffractometer	6422 independent reflections
Radiation source: fine-focus sealed tube	5830 reflections with *I* > 2σ(*I*)
graphite	*R*_int_ = 0.035
φ and ω scans	θ_max_ = 69.7°, θ_min_ = 2.5°
Absorption correction: numerical (*SAINT*; Bruker, 2007)	*h* = −10→10
*T*_min_ = 0.086, *T*_max_ = 0.348	*k* = −14→14
19130 measured reflections	*l* = −21→21

### Refinement

**Table:**

Refinement on *F*^2^	Primary atom site location: structure-invariant direct methods
Least-squares matrix: full	Secondary atom site location: difference Fourier map
*R*[*F*^2^ > 2σ(*F*^2^)] = 0.036	Hydrogen site location: inferred from neighbouring sites
*wR*(*F*^2^) = 0.093	H-atom parameters constrained
*S* = 1.05	*w* = 1/[σ^2^(*F*_o_^2^) + (0.0539*P*)^2^ + 2.2192*P*] where *P* = (*F*_o_^2^ + 2*F*_c_^2^)/3
6422 reflections	(Δ/σ)_max_ = 0.043
534 parameters	Δρ_max_ = 1.46 e Å^−^^3^
3 restraints	Δρ_min_ = −0.88 e Å^−^^3^

### Special details

**Table:**

Geometry. All e.s.d.'s (except the e.s.d. in the dihedral angle between two l.s. planes) are estimated using the full covariance matrix. The cell e.s.d.'s are taken into account individually in the estimation of e.s.d.'s in distances, angles and torsion angles; correlations between e.s.d.'s in cell parameters are only used when they are defined by crystal symmetry. An approximate (isotropic) treatment of cell e.s.d.'s is used for estimating e.s.d.'s involving l.s. planes.
Refinement. Refinement of *F*^2^ against ALL reflections. The weighted *R*-factor *wR* and goodness of fit *S* are based on *F*^2^, conventional *R*-factors *R* are based on *F*, with *F* set to zero for negative *F*^2^. The threshold expression of *F*^2^ > σ(*F*^2^) is used only for calculating *R*-factors(gt) *etc*. and is not relevant to the choice of reflections for refinement. *R*-factors based on *F*^2^ are statistically about twice as large as those based on *F*, and *R*- factors based on ALL data will be even larger.

### Fractional atomic coordinates and isotropic or equivalent isotropic displacement parameters (Å^2^)

**Table:**

	*x*	*y*	*z*	*U*_iso_*/*U*_eq_	Occ. (<1)
Pd1	0.88403 (6)	0.79572 (4)	0.19133 (3)	0.02224 (17)	
P1	0.7299 (2)	0.67699 (15)	0.26210 (10)	0.0200 (4)	
C1	0.8178 (10)	0.9157 (7)	0.2617 (5)	0.0305 (17)	
H1A	0.9104	0.9464	0.2697	0.046*	
H1B	0.7698	0.8781	0.3103	0.046*	
H1C	0.7415	0.9801	0.2387	0.046*	
N2	1.0136 (8)	0.9151 (6)	0.1220 (4)	0.0279 (14)	
C3	1.0826 (9)	0.9841 (7)	0.0885 (4)	0.0251 (15)	
C4	1.1735 (10)	1.0729 (7)	0.0447 (5)	0.0316 (17)	
H4A	1.1014	1.1415	0.0232	0.047*	
H4B	1.2423	1.0406	0.0039	0.047*	
H4C	1.2374	1.0960	0.0780	0.047*	
N5	0.9618 (7)	0.6603 (6)	0.1231 (3)	0.0251 (13)	
C6	1.1173 (9)	0.6238 (7)	0.1095 (4)	0.0283 (16)	
H6	1.1877	0.6752	0.1122	0.034*	
C7	1.1789 (10)	0.5154 (8)	0.0918 (4)	0.0318 (17)	
H7	1.2893	0.4931	0.0820	0.038*	
C8	1.0768 (10)	0.4392 (8)	0.0886 (5)	0.0322 (17)	
H8	1.1161	0.3626	0.0788	0.039*	
C9	0.9161 (10)	0.4774 (7)	0.0999 (4)	0.0295 (16)	
H9	0.8441	0.4278	0.0962	0.035*	
C10	0.8615 (9)	0.5875 (7)	0.1165 (4)	0.0253 (15)	
C11	0.6888 (9)	0.6356 (7)	0.1274 (4)	0.0266 (16)	
H11A	0.6704	0.7160	0.0980	0.032*	
H11B	0.6292	0.5872	0.1068	0.032*	
O12	0.6279 (6)	0.6381 (4)	0.2067 (3)	0.0228 (10)	
C13	0.5605 (8)	0.7290 (6)	0.3291 (4)	0.0207 (14)	
C14	0.4259 (9)	0.7999 (6)	0.2991 (4)	0.0225 (14)	
C15	0.2888 (9)	0.8282 (6)	0.3458 (4)	0.0255 (15)	
H15	0.1999	0.8755	0.3243	0.031*	
C16	0.2801 (9)	0.7889 (7)	0.4226 (4)	0.0272 (16)	
C17	0.4112 (9)	0.7274 (6)	0.4540 (4)	0.0259 (15)	
H17	0.4076	0.7050	0.5074	0.031*	
C18	0.5494 (9)	0.6975 (6)	0.4090 (4)	0.0243 (15)	
C19	0.4112 (9)	0.8531 (7)	0.2163 (4)	0.0259 (15)	
F20	0.3435 (5)	0.7874 (4)	0.1823 (2)	0.0305 (10)	
F21	0.5456 (5)	0.8716 (4)	0.1750 (2)	0.0297 (10)	
F22	0.3187 (6)	0.9577 (4)	0.2110 (3)	0.0355 (11)	
C23	0.1304 (10)	0.8149 (8)	0.4732 (5)	0.0348 (18)	
F24	0.0112 (6)	0.8704 (6)	0.4348 (3)	0.0530 (15)	
F25	0.1458 (8)	0.8773 (8)	0.5225 (5)	0.084 (3)	
F26	0.0838 (7)	0.7169 (6)	0.5117 (4)	0.0626 (18)	
C27	0.6838 (10)	0.6287 (7)	0.4516 (4)	0.0306 (17)	
F28	0.6692 (6)	0.6487 (5)	0.5223 (3)	0.0436 (13)	
F29	0.6908 (6)	0.5127 (4)	0.4572 (3)	0.0388 (11)	
F30	0.8222 (5)	0.6545 (4)	0.4194 (3)	0.0348 (10)	
C31	0.8327 (8)	0.5257 (6)	0.2986 (4)	0.0207 (14)	
C32	0.7605 (9)	0.4264 (7)	0.2976 (4)	0.0260 (15)	
C33	0.8487 (10)	0.3176 (7)	0.2930 (5)	0.0304 (17)	
H33	0.7981	0.2540	0.2903	0.036*	
C34	1.0100 (10)	0.3012 (7)	0.2922 (5)	0.0338 (19)	
C35	1.0823 (9)	0.3903 (7)	0.3014 (5)	0.0318 (17)	
H35	1.1921	0.3765	0.3054	0.038*	
C36	0.9955 (9)	0.5019 (7)	0.3047 (4)	0.0263 (15)	
C37	0.5854 (9)	0.4261 (7)	0.3027 (4)	0.0275 (16)	
F38	0.4982 (5)	0.5129 (4)	0.3348 (2)	0.0268 (9)	
F39	0.5506 (6)	0.3264 (4)	0.3475 (3)	0.0340 (10)	
F40	0.5333 (5)	0.4294 (4)	0.2367 (3)	0.0334 (10)	
C41	1.1054 (12)	0.1834 (8)	0.2817 (6)	0.047 (2)	
F42	1.0618 (9)	0.0980 (5)	0.3320 (4)	0.076 (2)	
F43	1.0811 (9)	0.1564 (6)	0.2159 (4)	0.074 (2)	
F44	1.2567 (7)	0.1820 (6)	0.2777 (5)	0.076 (2)	
C45	1.1009 (9)	0.5837 (7)	0.3185 (5)	0.0292 (16)	
F46	1.1596 (5)	0.5423 (4)	0.3843 (3)	0.0351 (11)	
F47	1.2226 (5)	0.5887 (5)	0.2645 (3)	0.0364 (11)	
F48	1.0343 (5)	0.6941 (4)	0.3209 (3)	0.0317 (10)	
Sb1	0.60710 (6)	0.23126 (4)	0.07962 (3)	0.02735 (18)	
F11	0.5822 (6)	0.3936 (4)	0.0756 (3)	0.0385 (11)	
F12	0.6312 (8)	0.2064 (5)	0.1830 (3)	0.0544 (15)	
F13	0.6346 (8)	0.0698 (5)	0.0829 (4)	0.0577 (17)	
F14	0.5886 (7)	0.2566 (6)	−0.0242 (3)	0.0483 (14)	
F15	0.8255 (6)	0.2283 (5)	0.0575 (3)	0.0467 (13)	
F16	0.3891 (6)	0.2383 (5)	0.0997 (4)	0.0528 (15)	
Cl1	0.5356 (9)	0.0251 (5)	0.4168 (5)	0.153 (3)	
C50	0.390 (4)	0.059 (2)	0.481 (2)	0.106 (13)	0.50
H50A	0.2931	0.0323	0.4729	0.127*	0.50
H50B	0.3670	0.1448	0.4795	0.127*	0.50

### Atomic displacement parameters (Å^2^)

**Table:**

	*U*^11^	*U*^22^	*U*^33^	*U*^12^	*U*^13^	*U*^23^
Pd1	0.0200 (3)	0.0197 (3)	0.0254 (3)	−0.0038 (2)	−0.0024 (2)	0.0004 (2)
P1	0.0169 (8)	0.0179 (8)	0.0230 (8)	−0.0007 (6)	−0.0016 (6)	−0.0008 (7)
C1	0.035 (4)	0.022 (4)	0.035 (4)	−0.004 (3)	−0.006 (3)	−0.004 (3)
N2	0.025 (3)	0.027 (3)	0.030 (3)	−0.006 (3)	−0.003 (3)	0.000 (3)
C3	0.023 (4)	0.025 (4)	0.027 (4)	−0.002 (3)	−0.004 (3)	−0.004 (3)
C4	0.033 (4)	0.028 (4)	0.033 (4)	−0.011 (3)	0.000 (3)	0.000 (3)
N5	0.025 (3)	0.025 (3)	0.023 (3)	−0.005 (3)	0.001 (2)	0.000 (3)
C6	0.024 (4)	0.033 (4)	0.026 (4)	−0.006 (3)	0.003 (3)	−0.001 (3)
C7	0.023 (4)	0.039 (5)	0.029 (4)	−0.003 (3)	0.005 (3)	−0.003 (3)
C8	0.034 (4)	0.030 (4)	0.030 (4)	0.000 (3)	0.002 (3)	−0.007 (3)
C9	0.030 (4)	0.033 (4)	0.026 (4)	−0.008 (3)	0.003 (3)	−0.007 (3)
C10	0.026 (4)	0.030 (4)	0.020 (3)	−0.007 (3)	0.002 (3)	−0.004 (3)
C11	0.024 (4)	0.034 (4)	0.021 (3)	−0.006 (3)	−0.002 (3)	−0.002 (3)
O12	0.018 (2)	0.025 (3)	0.024 (2)	−0.003 (2)	−0.0003 (19)	−0.004 (2)
C13	0.017 (3)	0.016 (3)	0.027 (3)	−0.001 (3)	−0.001 (3)	−0.003 (3)
C14	0.024 (4)	0.015 (3)	0.029 (4)	−0.003 (3)	−0.006 (3)	−0.003 (3)
C15	0.024 (4)	0.019 (4)	0.033 (4)	0.000 (3)	−0.006 (3)	−0.005 (3)
C16	0.028 (4)	0.020 (4)	0.034 (4)	−0.004 (3)	0.003 (3)	−0.010 (3)
C17	0.033 (4)	0.019 (4)	0.025 (3)	−0.003 (3)	−0.002 (3)	−0.003 (3)
C18	0.025 (4)	0.020 (4)	0.028 (4)	−0.003 (3)	−0.003 (3)	−0.004 (3)
C19	0.023 (4)	0.024 (4)	0.028 (4)	0.001 (3)	−0.003 (3)	−0.003 (3)
F20	0.028 (2)	0.036 (3)	0.028 (2)	−0.0051 (19)	−0.0070 (18)	−0.0070 (19)
F21	0.025 (2)	0.029 (2)	0.030 (2)	−0.0036 (18)	−0.0027 (18)	0.0046 (19)
F22	0.038 (3)	0.028 (2)	0.035 (2)	0.011 (2)	−0.007 (2)	−0.002 (2)
C23	0.027 (4)	0.039 (5)	0.035 (4)	0.003 (4)	0.001 (3)	−0.008 (4)
F24	0.029 (3)	0.067 (4)	0.046 (3)	0.014 (3)	0.006 (2)	0.001 (3)
F25	0.046 (4)	0.136 (7)	0.091 (5)	−0.017 (4)	0.020 (4)	−0.085 (6)
F26	0.043 (3)	0.057 (4)	0.065 (4)	0.003 (3)	0.024 (3)	0.012 (3)
C27	0.032 (4)	0.028 (4)	0.026 (4)	0.005 (3)	−0.004 (3)	0.000 (3)
F28	0.043 (3)	0.057 (3)	0.026 (2)	0.013 (2)	−0.010 (2)	−0.008 (2)
F29	0.039 (3)	0.028 (2)	0.040 (3)	0.006 (2)	−0.004 (2)	0.006 (2)
F30	0.025 (2)	0.043 (3)	0.036 (2)	−0.002 (2)	−0.0054 (19)	−0.006 (2)
C31	0.018 (3)	0.019 (3)	0.021 (3)	0.000 (3)	0.002 (3)	0.001 (3)
C32	0.022 (4)	0.025 (4)	0.027 (4)	−0.003 (3)	0.005 (3)	−0.002 (3)
C33	0.030 (4)	0.023 (4)	0.034 (4)	−0.004 (3)	0.010 (3)	−0.002 (3)
C34	0.027 (4)	0.024 (4)	0.042 (5)	0.003 (3)	0.011 (3)	0.000 (4)
C35	0.020 (4)	0.028 (4)	0.039 (4)	0.002 (3)	0.004 (3)	0.004 (3)
C36	0.023 (4)	0.025 (4)	0.027 (4)	−0.001 (3)	0.001 (3)	0.001 (3)
C37	0.026 (4)	0.022 (4)	0.034 (4)	−0.005 (3)	0.004 (3)	−0.006 (3)
F38	0.019 (2)	0.025 (2)	0.035 (2)	−0.0022 (17)	0.0041 (17)	−0.0083 (19)
F39	0.030 (2)	0.025 (2)	0.044 (3)	−0.0108 (19)	0.010 (2)	−0.004 (2)
F40	0.031 (2)	0.037 (3)	0.035 (2)	−0.010 (2)	−0.0001 (19)	−0.012 (2)
C41	0.037 (5)	0.028 (5)	0.062 (6)	0.007 (4)	0.020 (4)	−0.005 (4)
F42	0.086 (5)	0.026 (3)	0.089 (5)	0.013 (3)	0.036 (4)	0.006 (3)
F43	0.087 (5)	0.050 (4)	0.076 (5)	0.019 (4)	0.004 (4)	−0.028 (4)
F44	0.034 (3)	0.043 (3)	0.148 (8)	0.012 (3)	0.005 (4)	−0.033 (4)
C45	0.019 (4)	0.031 (4)	0.032 (4)	−0.002 (3)	−0.003 (3)	0.005 (3)
F46	0.029 (2)	0.040 (3)	0.033 (2)	−0.002 (2)	−0.0103 (19)	0.004 (2)
F47	0.023 (2)	0.048 (3)	0.037 (3)	−0.012 (2)	0.0014 (19)	−0.002 (2)
F48	0.026 (2)	0.025 (2)	0.043 (3)	−0.0037 (18)	−0.0132 (19)	0.002 (2)
Sb1	0.0290 (3)	0.0234 (3)	0.0279 (3)	−0.0051 (2)	0.0026 (2)	−0.0033 (2)
F11	0.035 (3)	0.024 (2)	0.056 (3)	−0.004 (2)	−0.004 (2)	−0.005 (2)
F12	0.083 (4)	0.045 (3)	0.028 (3)	0.007 (3)	−0.006 (3)	−0.004 (2)
F13	0.083 (5)	0.024 (3)	0.063 (4)	−0.013 (3)	0.017 (3)	−0.013 (3)
F14	0.050 (3)	0.068 (4)	0.030 (3)	−0.013 (3)	−0.007 (2)	−0.010 (3)
F15	0.025 (3)	0.049 (3)	0.060 (3)	0.001 (2)	−0.001 (2)	−0.004 (3)
F16	0.029 (3)	0.055 (3)	0.074 (4)	−0.018 (3)	0.015 (3)	−0.018 (3)
Cl1	0.147 (5)	0.104 (4)	0.221 (8)	−0.059 (4)	−0.011 (5)	−0.035 (5)
C50	0.09 (2)	0.044 (14)	0.19 (4)	−0.035 (15)	0.02 (2)	−0.05 (2)

### Geometric parameters (Å, °)

**Table:**

Pd1—C1	2.047 (8)	C19—F21	1.327 (9)
Pd1—N2	2.079 (7)	C19—F20	1.336 (9)
Pd1—N5	2.170 (6)	C19—F22	1.340 (9)
Pd1—P1	2.2146 (18)	C23—F25	1.301 (11)
P1—O12	1.609 (5)	C23—F26	1.328 (11)
P1—C13	1.870 (7)	C23—F24	1.328 (10)
P1—C31	1.883 (7)	C27—F30	1.324 (10)
C1—H1A	0.9800	C27—F28	1.335 (9)
C1—H1B	0.9800	C27—F29	1.344 (10)
C1—H1C	0.9800	C31—C36	1.408 (11)
N2—C3	1.128 (10)	C31—C32	1.432 (10)
C3—C4	1.467 (11)	C32—C33	1.383 (11)
C4—H4A	0.9800	C32—C37	1.513 (11)
C4—H4B	0.9800	C33—C34	1.379 (12)
C4—H4C	0.9800	C33—H33	0.9500
N5—C6	1.347 (10)	C34—C35	1.367 (12)
N5—C10	1.363 (10)	C34—C41	1.517 (11)
C6—C7	1.377 (12)	C35—C36	1.403 (11)
C6—H6	0.9500	C35—H35	0.9500
C7—C8	1.388 (12)	C36—C45	1.523 (11)
C7—H7	0.9500	C37—F40	1.329 (9)
C8—C9	1.388 (12)	C37—F38	1.341 (9)
C8—H8	0.9500	C37—F39	1.349 (9)
C9—C10	1.378 (11)	C41—F42	1.294 (11)
C9—H9	0.9500	C41—F44	1.305 (12)
C10—C11	1.507 (11)	C41—F43	1.347 (14)
C11—O12	1.467 (8)	C45—F46	1.332 (9)
C11—H11A	0.9900	C45—F48	1.334 (9)
C11—H11B	0.9900	C45—F47	1.340 (9)
C13—C14	1.418 (10)	Sb1—F13	1.868 (5)
C13—C18	1.418 (10)	Sb1—F16	1.870 (5)
C14—C15	1.394 (11)	Sb1—F14	1.873 (5)
C14—C19	1.523 (10)	Sb1—F12	1.876 (5)
C15—C16	1.374 (11)	Sb1—F11	1.879 (5)
C15—H15	0.9500	Sb1—F11	1.879 (5)
C16—C17	1.367 (11)	Sb1—F15	1.881 (5)
C16—C23	1.506 (11)	Cl1—C50	1.65 (2)
C17—C18	1.388 (11)	C50—H50A	0.9900
C17—H17	0.9500	C50—H50B	0.9900
C18—C27	1.510 (11)		
			
C1—Pd1—N2	87.5 (3)	F25—C23—F24	107.8 (8)
C1—Pd1—N5	176.1 (3)	F26—C23—F24	105.3 (7)
N2—Pd1—N5	94.4 (2)	F25—C23—C16	112.5 (7)
C1—Pd1—P1	91.4 (2)	F26—C23—C16	111.1 (7)
N2—Pd1—P1	175.69 (19)	F24—C23—C16	112.6 (7)
N5—Pd1—P1	86.99 (17)	F30—C27—F28	106.8 (7)
O12—P1—C13	96.7 (3)	F30—C27—F29	107.1 (6)
O12—P1—C31	96.4 (3)	F28—C27—F29	106.2 (7)
C13—P1—C31	112.8 (3)	F30—C27—C18	112.7 (7)
O12—P1—Pd1	107.5 (2)	F28—C27—C18	111.7 (6)
C13—P1—Pd1	122.6 (2)	F29—C27—C18	112.0 (7)
C31—P1—Pd1	115.0 (2)	C36—C31—C32	115.5 (7)
Pd1—C1—H1A	109.5	C36—C31—P1	122.0 (6)
Pd1—C1—H1B	109.5	C32—C31—P1	119.5 (6)
H1A—C1—H1B	109.5	C33—C32—C31	121.6 (7)
Pd1—C1—H1C	109.5	C33—C32—C37	112.9 (7)
H1A—C1—H1C	109.5	C31—C32—C37	125.5 (7)
H1B—C1—H1C	109.5	C34—C33—C32	120.3 (8)
C3—N2—Pd1	175.1 (6)	C34—C33—H33	119.9
N2—C3—C4	179.5 (9)	C32—C33—H33	119.9
C3—C4—H4A	109.5	C35—C34—C33	120.0 (8)
C3—C4—H4B	109.5	C35—C34—C41	120.6 (8)
H4A—C4—H4B	109.5	C33—C34—C41	119.3 (8)
C3—C4—H4C	109.5	C34—C35—C36	120.5 (8)
H4A—C4—H4C	109.5	C34—C35—H35	119.8
H4B—C4—H4C	109.5	C36—C35—H35	119.8
C6—N5—C10	118.0 (7)	C35—C36—C31	121.4 (7)
C6—N5—Pd1	118.4 (5)	C35—C36—C45	110.3 (7)
C10—N5—Pd1	119.8 (5)	C31—C36—C45	128.2 (7)
N5—C6—C7	123.0 (7)	F40—C37—F38	108.0 (6)
N5—C6—H6	118.5	F40—C37—F39	106.2 (6)
C7—C6—H6	118.5	F38—C37—F39	105.4 (6)
C6—C7—C8	118.8 (7)	F40—C37—C32	114.0 (6)
C6—C7—H7	120.6	F38—C37—C32	113.5 (6)
C8—C7—H7	120.6	F39—C37—C32	109.1 (7)
C9—C8—C7	118.6 (8)	F42—C41—F44	111.0 (9)
C9—C8—H8	120.7	F42—C41—F43	103.6 (9)
C7—C8—H8	120.7	F44—C41—F43	105.5 (8)
C10—C9—C8	119.8 (7)	F42—C41—C34	112.8 (7)
C10—C9—H9	120.1	F44—C41—C34	112.9 (8)
C8—C9—H9	120.1	F43—C41—C34	110.3 (9)
N5—C10—C9	121.5 (7)	F46—C45—F48	106.3 (7)
N5—C10—C11	115.9 (7)	F46—C45—F47	107.4 (6)
C9—C10—C11	122.5 (7)	F48—C45—F47	106.3 (6)
O12—C11—C10	113.5 (6)	F46—C45—C36	109.5 (6)
O12—C11—H11A	108.9	F48—C45—C36	116.7 (6)
C10—C11—H11A	108.9	F47—C45—C36	110.2 (7)
O12—C11—H11B	108.9	F13—Sb1—F16	91.2 (3)
C10—C11—H11B	108.9	F13—Sb1—F14	90.0 (3)
H11A—C11—H11B	107.7	F16—Sb1—F14	89.7 (3)
C11—O12—P1	120.4 (4)	F13—Sb1—F12	90.2 (3)
C14—C13—C18	115.7 (6)	F16—Sb1—F12	91.8 (3)
C14—C13—P1	118.6 (5)	F14—Sb1—F12	178.5 (3)
C18—C13—P1	125.5 (5)	F13—Sb1—F11	179.2 (3)
C15—C14—C13	121.0 (7)	F16—Sb1—F11	89.6 (2)
C15—C14—C19	112.6 (6)	F14—Sb1—F11	89.8 (3)
C13—C14—C19	126.3 (7)	F12—Sb1—F11	90.0 (2)
C16—C15—C14	121.0 (7)	F13—Sb1—F11	179.2 (3)
C16—C15—H15	119.5	F16—Sb1—F11	89.6 (2)
C14—C15—H15	119.5	F14—Sb1—F11	89.8 (3)
C17—C16—C15	119.4 (7)	F12—Sb1—F11	90.0 (2)
C17—C16—C23	119.1 (7)	F11—Sb1—F11	0.0 (3)
C15—C16—C23	121.5 (7)	F13—Sb1—F15	90.1 (3)
C16—C17—C18	120.8 (7)	F16—Sb1—F15	178.4 (3)
C16—C17—H17	119.6	F14—Sb1—F15	89.3 (3)
C18—C17—H17	119.6	F12—Sb1—F15	89.2 (3)
C17—C18—C13	121.7 (7)	F11—Sb1—F15	89.1 (2)
C17—C18—C27	114.9 (7)	F11—Sb1—F15	89.1 (2)
C13—C18—C27	123.4 (7)	F11—F11—Sb1	0(10)
F21—C19—F20	107.6 (6)	C50—Cl1—C50^i^	74.4 (19)
F21—C19—F22	105.9 (6)	Cl1—C50—Cl1^i^	105.6 (19)
F20—C19—F22	106.3 (6)	Cl1—C50—H50A	110.6
F21—C19—C14	114.9 (6)	Cl1^i^—C50—H50A	110.6
F20—C19—C14	111.7 (6)	Cl1—C50—H50B	110.6
F22—C19—C14	109.8 (6)	Cl1^i^—C50—H50B	110.6
F25—C23—F26	107.0 (8)	H50A—C50—H50B	108.7
			
C1—Pd1—P1—O12	130.9 (3)	C15—C14—C19—F21	153.5 (6)
N2—Pd1—P1—O12	57 (3)	C13—C14—C19—F21	−26.5 (10)
N5—Pd1—P1—O12	−52.7 (3)	C15—C14—C19—F20	−83.6 (8)
C1—Pd1—P1—C13	20.6 (4)	C13—C14—C19—F20	96.5 (8)
N2—Pd1—P1—C13	−54 (3)	C15—C14—C19—F22	34.2 (9)
N5—Pd1—P1—C13	−163.0 (3)	C13—C14—C19—F22	−145.7 (7)
C1—Pd1—P1—C31	−123.1 (3)	C17—C16—C23—F25	60.7 (11)
N2—Pd1—P1—C31	163 (3)	C15—C16—C23—F25	−117.5 (9)
N5—Pd1—P1—C31	53.3 (3)	C17—C16—C23—F26	−59.3 (10)
C1—Pd1—N2—C3	15 (8)	C15—C16—C23—F26	122.5 (8)
N5—Pd1—N2—C3	−162 (8)	C17—C16—C23—F24	−177.2 (7)
P1—Pd1—N2—C3	89 (8)	C15—C16—C23—F24	4.6 (11)
Pd1—N2—C3—C4	132 (100)	C17—C18—C27—F30	−145.4 (7)
C1—Pd1—N5—C6	−62 (4)	C13—C18—C27—F30	35.2 (10)
N2—Pd1—N5—C6	56.6 (6)	C17—C18—C27—F28	−25.3 (10)
P1—Pd1—N5—C6	−127.5 (6)	C13—C18—C27—F28	155.4 (7)
C1—Pd1—N5—C10	96 (4)	C17—C18—C27—F29	93.7 (8)
N2—Pd1—N5—C10	−145.5 (6)	C13—C18—C27—F29	−85.6 (9)
P1—Pd1—N5—C10	30.4 (5)	O12—P1—C31—C36	134.8 (6)
C10—N5—C6—C7	−2.2 (11)	C13—P1—C31—C36	−125.2 (6)
Pd1—N5—C6—C7	156.1 (6)	Pd1—P1—C31—C36	22.1 (7)
N5—C6—C7—C8	−0.7 (12)	O12—P1—C31—C32	−25.0 (6)
C6—C7—C8—C9	2.9 (12)	C13—P1—C31—C32	75.1 (6)
C7—C8—C9—C10	−2.1 (12)	Pd1—P1—C31—C32	−137.7 (5)
C6—N5—C10—C9	3.0 (11)	C36—C31—C32—C33	−8.2 (11)
Pd1—N5—C10—C9	−155.0 (6)	P1—C31—C32—C33	152.8 (6)
C6—N5—C10—C11	−176.2 (6)	C36—C31—C32—C37	170.7 (7)
Pd1—N5—C10—C11	25.8 (8)	P1—C31—C32—C37	−28.2 (10)
C8—C9—C10—N5	−0.8 (12)	C31—C32—C33—C34	2.7 (12)
C8—C9—C10—C11	178.3 (7)	C37—C32—C33—C34	−176.4 (7)
N5—C10—C11—O12	−75.3 (8)	C32—C33—C34—C35	4.6 (13)
C9—C10—C11—O12	105.5 (8)	C32—C33—C34—C41	−175.9 (8)
C10—C11—O12—P1	40.9 (8)	C33—C34—C35—C36	−5.8 (13)
C13—P1—O12—C11	154.3 (5)	C41—C34—C35—C36	174.7 (8)
C31—P1—O12—C11	−91.7 (6)	C34—C35—C36—C31	−0.3 (12)
Pd1—P1—O12—C11	27.0 (6)	C34—C35—C36—C45	177.6 (7)
O12—P1—C13—C14	−45.0 (6)	C32—C31—C36—C35	7.0 (11)
C31—P1—C13—C14	−144.8 (5)	P1—C31—C36—C35	−153.5 (6)
Pd1—P1—C13—C14	70.7 (6)	C32—C31—C36—C45	−170.4 (7)
O12—P1—C13—C18	130.7 (6)	P1—C31—C36—C45	29.1 (11)
C31—P1—C13—C18	30.9 (7)	C33—C32—C37—F40	−79.3 (9)
Pd1—P1—C13—C18	−113.5 (6)	C31—C32—C37—F40	101.7 (9)
C18—C13—C14—C15	−4.7 (10)	C33—C32—C37—F38	156.4 (7)
P1—C13—C14—C15	171.4 (5)	C31—C32—C37—F38	−22.6 (11)
C18—C13—C14—C19	175.2 (7)	C33—C32—C37—F39	39.2 (9)
P1—C13—C14—C19	−8.6 (9)	C31—C32—C37—F39	−139.8 (7)
C13—C14—C15—C16	0.4 (11)	C35—C34—C41—F42	122.7 (11)
C19—C14—C15—C16	−179.5 (7)	C33—C34—C41—F42	−56.8 (14)
C14—C15—C16—C17	4.3 (11)	C35—C34—C41—F44	−4.1 (14)
C14—C15—C16—C23	−177.5 (7)	C33—C34—C41—F44	176.3 (9)
C15—C16—C17—C18	−4.5 (11)	C35—C34—C41—F43	−121.9 (10)
C23—C16—C17—C18	177.2 (7)	C33—C34—C41—F43	58.5 (11)
C16—C17—C18—C13	0.0 (11)	C35—C36—C45—F46	−61.1 (8)
C16—C17—C18—C27	−179.4 (7)	C31—C36—C45—F46	116.6 (8)
C14—C13—C18—C17	4.5 (10)	C35—C36—C45—F48	178.2 (7)
P1—C13—C18—C17	−171.3 (6)	C31—C36—C45—F48	−4.2 (12)
C14—C13—C18—C27	−176.1 (7)	C35—C36—C45—F47	56.8 (8)
P1—C13—C18—C27	8.0 (10)	C31—C36—C45—F47	−125.5 (8)

Symmetry codes:  (i) −*x*+1, −*y*, −*z*+1.

### Hydrogen-bond geometry (Å, °)

**Table:**

*D*—H···*A*	*D*—H	H···*A*	*D*···*A*	*D*—H···*A*
C4—H4C···F16^ii^	0.98	2.44	3.310 (10)	148
C11—H11B···F11	0.99	2.58	3.489 (9)	154
C9—H9···F11	0.95	2.47	3.345 (9)	152

Symmetry codes: (ii) *x*+1, *y*+1, *z*.
